# Modulating Brain Networks With Transcranial Magnetic Stimulation Over the Primary Motor Cortex: A Concurrent TMS/fMRI Study

**DOI:** 10.3389/fnhum.2020.00031

**Published:** 2020-02-14

**Authors:** JeYoung Jung, Andreas Bungert, Richard Bowtell, Stephen R. Jackson

**Affiliations:** ^1^School of Psychology, University of Nottingham, Nottingham, United Kingdom; ^2^Department of Brain and Cognitive Engineering, Korea University, Seoul, South Korea; ^3^Sir Peter Mansfield Magnetic Resonance Centre, University of Nottingham, Nottingham, United Kingdom

**Keywords:** primary motor cortex, concurrent TMS/fMRI, functional brain networks, insular, rolandic operculum, bodily self-consciousness

## Abstract

Stimulating the primary motor cortex (M1) using transcranial magnetic stimulation (TMS) causes unique multisensory experience such as the targeted muscle activity, afferent/reafferent sensory feedback, tactile sensation over the scalp and “click” sound. Although the human M1 has been intensively investigated using TMS, the experience of the M1 stimulation has not been elucidated at the whole brain. Here, using concurrent TMS/fMRI, we investigated the acute effect of the M1 stimulation of functional brain networks during task and at rest. A short train of 1 Hz TMS pulses applied to individuals’ hand area in the M1 during motor execution or at rest. Employing the independent component analysis (ICA), we showed the M1 stimulation decreased the motor networks activity when the networks were engaged in the task and increased the deactivation of networks when the networks were not involved in the ongoing task. The M1 stimulation induced the activation in the key networks involved in bodily self-consciousness (BSC) including the insular and rolandic operculum systems regardless of states. The degree of activation in these networks was prominent at rest compared to task conditions, showing the state-dependent TMS effect. Furthermore, we demonstrated that the M1 stimulation modulated other domain-general networks such as the default mode network (DMN) and attention network and the inter-network connectivity between these networks. Our results showed that the M1 stimulation induced the widespread changes in the brain at the targeted system as well as non-motor, remote brain networks, specifically related to the BSC. Our findings shed light on understanding the neural mechanism of the complex and multisensory experience of the M1 stimulation.

## Introduction

Transcranial magnetic stimulation (TMS) is a non-invasive brain stimulation technique that has been widely used to investigate brain function. TMS induces a brief magnetic field at the scalp which can then transiently modulate brain electrical activity leading to behavioral changes (Walsh and Cowey, 2000). In the human brain, the primary motor cortex (M1) has been intensively investigated using TMS to understand the neural mechanism of the motor system (Wasserman et al., 2008). Different from other regions, the M1 stimulation elicits motor-evoked potential (MEP), a measurement of corticospinal excitability, accompanying with a unique sensation at the targeted hand region or hand twitch with suprathreshold intensity. Studies on the M1 stimulation have focused on the MEP linking to neurophysiological mechanism of the motor system but the whole experience of the M1 stimulation (e.g., afferent/reafferent sensory feedback, tactile sensation over the scalp, and “click” sound) has been disregarded. Thus, it is not clear how the experience of the M1 TMS influences the neural processing at the whole brain.

The M1 TMS affects both the targeted area and functionally connected remote areas (Ferbert et al., 1992; Di Lazzaro et al., 1999; Koch et al., 2006). To demonstrate it, there have been a number of attempts to combine TMS with other neuroimaging techniques (e.g., fMRI). Bohning et al. (1998) first demonstrated that TMS induced blood-oxygenation-level-dependent (BOLD) changes at the target site and a number of remote areas when TMS was applied on the M1. Since the first successful demonstration of the combination of TMS and fMRI, several studies have been published using concurrent TMS/fMRI (Bohning et al., 1999, 2000a,b, 2003; Baudewig et al., 2001; Bestmann et al., 2003, 2004, 2005; Li et al., 2004; Denslow et al., 2005; Ruff et al., 2009). Initial studies to stimulate the M1 at rest demonstrated that TMS caused significant BOLD changes at the target region, functionally connected cortical and subcortical motor regions as well as non-motor regions such as auditory cortex, insular, frontal and parietal regions (Bohning et al., 1999, Bohning et al., 2000a,b; Bestmann et al., 2003, 2004; Denslow et al., 2005). Bestmann et al. (2008) stimulated the left premotor cortex (PMC) during a motor execution task (grip vs. no-grip) and demonstrated the state-dependent TMS effect in the contralateral M1 and PMC. These findings suggest that: (1) TMS induces neural changes across the whole brain including the target region and other remote areas; and (2) the current state of a targeted neural system influences the effect of TMS. However, studies have focused on the motor system at the regional activity level, disregarding other multisensory processing evoked by the M1 TMS. In addition, it remains unclear how TMS over the M1 modulates the brain at a network level, including the targeted network and other functional neural systems, depending on the current state of the network.

Here, we employed concurrent TMS/fMRI and independent component analysis (ICA)—a data-driven multivariate approach to decompose a mixed-signal into independent components (ICs; networks; Calhoun et al., 2001) to examine the effect of the M1 TMS at the whole-brain scale during two different states (task or rest). Specifically, we applied a short burst of the M1 stimulation during a motor execution task or at rest. A group of participants performed either unimanual or bimanual hand clenching with the left or right M1 stimulation. The other group received the left M1 stimulation without a task. As a control group, there was the vertex stimulation group at rest. In order to detect the neural changes related to the M1 TMS, we compared the local brain activity and network measurements driven from the ICA including the degree of network activity and functional network connectivity (FNC) with and without the stimulation. Based on the previous studies, we hypothesized that the M1 TMS exerted the BOLD changes at the target site and the targeted network—the motor network (MN). In addition, we expected that the M1 TMS induced neural changes in the non-motor remote regions/networks associated with the multisensory experience of it such as afferent/reafferent sensory feedback compared to the vertex TMS. Furthermore, these changes would be state-dependent: the current state of the neural system would influence the degree of the TMS effects.

## Materials and Methods

### Participants

Thirty-six healthy subjects (nine males: mean age 26 ± 5 years, range 19–32 years) were recruited for this study. Subjects were allocated into three groups: the task M1 stimulation group, rest M1 stimulation group and rest vertex stimulation group as a control group. The task M1 stimulation group (12 healthy adults, three males: mean age 27 ± 3 years, range 20–32 years) performed a motor execution task with the M1 stimulation. The rest M1 stimulation group (12 healthy adults, three males; mean age 25 ± 3.1 years, range 19–30 years) received the M1 stimulation without a task. As a control group, we had the vertex stimulation group from a previously published study (Jung et al., 2016; 12 healthy adults, three males; mean age 25 ± 8.3 years, range 20–28 years) received the vertex stimulation at rest. It should be noted that the vertex group data was collected with the same TMS/MRI instrument and fMRI acquisition parameters with the current study. They were all right-handed. Handedness was assessed by the Edinburgh handedness inventory (mean score: 95 ± 12; Oldfield, 1971). All participants provided informed written consent in advance the experiment. This study was approved by the local ethics committee and performed in accordance with the Declaration of Helsinki.

### Experiment Design and Procedure

**Figure 1:**
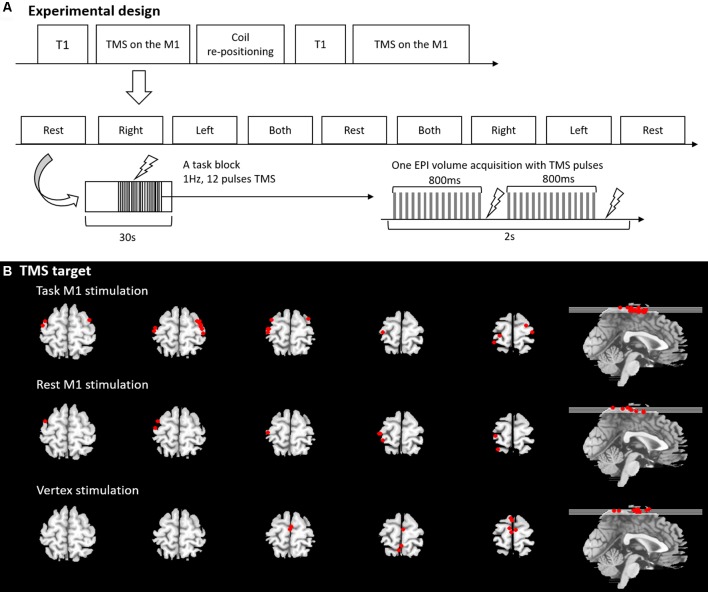
**(A)** Experimental design and procedure. **(B)** Transcranial magnetic stimulation (TMS) target position.

We used an fMRI block design with a block length of 30 s. During the task M1 stimulation, there were four sessions in the experiment and each session comprised of nine blocks (4 min 30 s; Figure 1). In each session, there were four experimental conditions. Participants were asked to perform simple hand clenching (HC) movements using their left (LHC), right (RHC), or both hands simultaneously (BHC), or else they were instructed to make no hand movements (rest). The hand clenching task required participants to continuously clench and unclench their hand while instruction words were displayed on the screen (left, right, both and rest). The order of task conditions was pseudorandomized and counterbalanced across the session. Each block was comprised of a TMS phase (11 s) and a No-TMS phase (19 s). The temporal onset of the TMS phase was randomized within each block. During the TMS phase, 11 pulses of 1 Hz TMS were delivered to the hand area of the left or right M1. Two sessions involved TMS delivered to the left M1 and two sessions involved TMS delivered to the right M1. After the first two sessions, the TMS coil was re-positioned to the contralateral M1. The order of the stimulation was counterbalanced across participants. The same fMRI paradigm with 18 blocks (9 min) was used for the rest M1 stimulation and the vertex stimulation without a task. Participant was asked to see the fixation on the screen.

Before the experiment, individual resting motor threshold (RMT) was measured for all participants outside of MRI scanner. In the MR scanner, participants’ hands were placed next to their body in a natural relaxed position. Each participant wore glasses with a prismatic mirror to view a projection screen on which visual stimuli were presented throughout the experiment. The task M1 stimulation group was asked to perform the HC task at a rate that they were comfortable with. Prior testing confirms that this was between 0.5 and 1 Hz. The instruction words for each condition were displayed on a projection screen at the foot of the scanner bed. The experimental design and procedure is illustrated in Figure 1A. The other groups were instructed to keep their limbs relaxed during the experiment.

### Magnetic Resonance Imaging

Functional MR images were acquired at the Magnetic Resonance Centre (University of Nottingham), using a Philips 3.0-Tesla scanner equipped with a 6-channel head coil to accommodate the TMS coil. Functional images were obtained using single-shot echo-planar imaging (EPI) sequence [repetition time (TR)/echo time (TE) = 2,000/35 ms, flip angle 90°, 30 slices, matrix = 64 × 64, 3 × 3 × 3 mm^3^ resolution]. Anatomical images were acquired using 3D MP-RAGE sequence (TR/TE = 8.278/2.3 ms, flip angle 8°, matrix = 192 × 192, 1 × 1 × 1 mm^3^ resolution) covering the whole head. During scanning, all participants wore ear-plugs with head cushioned by foam pads to prevent head-movement artifacts.

### Transcranial Magnetic Stimulation

A Magstim Rapid2 stimulator (Magstim, UK) was used to generate TMS pulses through an MR-compatible figure-of-eight coil (70 mm outer ring diameter). For the M1 stimulation, the coil was centered at the left or right-hand area. For the vertex stimulation, the coil was positioned at the vertex (Cz) using the international 10-20 system (Steinmetz et al., 1989). Individual RMTs were measured as follows: TMS pulses were applied to the M1 to identify the optimal site eliciting a muscle twitch in the left and the right FDI muscle and the TMS coil was oriented perpendicular to the central sulcus at a 45° angle from the mid-sagittal line approximately. Once a site was identified, the stimulator intensity was systematically varied and the RMT was defined as the minimum stimulator output that was required to induce an observable muscle twitch at that site for five out of 10 TMS pulses. Individual TMS intensity was 100% of RMT for the M1 stimulation. The vertex stimulation group received TMS pulses with 120% of RMT. The mean stimulator output corresponding to RMT was 75% for the right M1 stimulation (range 59–88%) and 75% for the left M1 stimulation (range 64–89%). In the rest M1 stimulation, the mean RMT was 72% ranging from 59% to 86%. The mean RMT of the vertex stimulation was 72% ranging from 59% to 86%. To hold the TMS coil, we used a plastic coil holder placed next to the MRI head coil.

### Synchronisation TMS and fMRI

Our previous study (Jung et al., 2016) showed a successful synchronization TMS and fMRI based on the findings of Shastri et al. (1999). In this experiment, the scanner sequence was programmed to split the acquisition of images in each volume into two separate packages. The first package was acquired for ~800 ms and the second package commenced collection 200 ms after the first package acquisition had ceased. We applied a TMS pulse 850 ms after the acquisition of the first slice in each package during the TMS phase of each block. In this way, TMS was applied at 850 ms and at 1,850 ms during each volume acquisition without distortion (Figure 1A). The synchronization of TMS pulse was carried out using an in-house Matlab programme written in Matlab (R2006b).

### TMS Coil Position and Target Site

The rubber ring around the TMS-coil was MR-visible for short echo-time (TE <10 ms) and it was used to verify the position of the TMS-coil relative to the subject. For this purpose magnetisation prepared rapid gradient echo (MP-RAGE) images were acquired for each position of the TMS-coil. These images covered the head of the subject and the rubber ring around the coil. Using in house-Matlab code, several points on the rubber ring were identified in the images. By fitting the shape of the rubber ring around the coil to these points in the image, the position of the TMS was determined. The location defined as coil position was the point where a virtual line perpendicular to the TMS-coil and through the center point of the TMS coil (where the two rings of the figure-of-eight meet each other) hits the brain surface. This position was translated into Montreal Neurological Institute (MNI) space by co-registering the MP-RAGE image to MNI space. TMS target sites were displayed in Figure 1B, Supplementary Table S1.

### Univariate Analysis

Statistical Parametric Mapping software (SPM12, Wellcome Department of Imaging Neuroscience, UK) was used for data analysis. All EPI images were realigned, co-registered with each individual’s anatomical image, spatially normalized to MNI space, and spatially smoothed using a Gaussian kernel (8 mm, Full-width half-maximal). The session with head movements exceeding more than 2 mm in x, y, z direction and 2° in rotation, was excluded from the analysis (only one session from the right M1 TMS was excluded). Head movement parameters were included within the analysis as regressor variables to exclude head movement-related variance.

A general linear model (GLM) was used to calculate individual contrasts. For the task M1 stimulation group, we defined a design matrix comprising four task conditions (LHC, RHC, BHC, and rest) and TMS phases (TMS and no-TMS). T-contrasts for each condition and TMS phase were established for all participants. In the group analysis, three-factorial ANOVA with the site of stimulation (left vs. right), task (LHC, RHC, and BHC), and TMS (TMS vs. no-TMS) was conducted and contrasts were entered into a set of one-sample *t*-tests for the three-movement conditions and TMS phases. For the rest M1 stimulation and vertex stimulation groups, a design matrix with TMS phases (TMS and no-TMS) was constructed. In the group level analysis, the contrast images were entered into one-sample *t*-tests. The statistical significance threshold was set to a height threshold of *p* < 0.005 uncorrected, at the voxel level and to that of *p* < 0.05 at the cluster level with at least 20 contiguous voxels after false-discovery rate (FDR) correction.

To explore the effect of TMS within the MN, regions of interest (ROIs) were defined as a 4 mm radius sphere based on the results of group-level analysis in the task M1 stimulation group. These included the M1 [Ml (−33, −24, 63), Mr (36, −15, 54)], PMC [PMCl (−50, −15, 37), PMCr (52, −12, 38)] and supplementary motor areas [SMA: SMAl (−3, −6, 48), SMAr (6, −6, 48)] in both hemispheres. ANOVAs with the site of stimulation (left vs. right), TMS (TMS vs. NoTMS), and hemisphere (left vs. right) as within-subject factors were conducted for each ROI, according to task conditions (LHC, RHC and BHC). To detect the effect of TMS at the each ROI, we performed the planned *t*-tests. Also, we performed a conjunction analysis to assess the general effect of TMS across the task conditions.

### Multivariate Analysis—Independent Component Analysis

ICA was used to estimate spatiotemporal functional networks from the data. ICA uses fluctuations in the fMRI data to separate the signal into maximally independent spatial maps or components, each explaining unique variance of the 4D fMRI data. Each component has a time course related to a coherent neural signal potentially associated with intrinsic brain networks, artifacts, or both.

ICA was performed using the group ICA of fMRI Toolbox (GIFT; Egolf et al., 2004). The pre-processed data was entered into the GIFT version 3.0b. The toolbox concatenates the individual data followed by the computation of subject-specific components and time course. Maximum Description Length (MDL) and Akaike’s criteria were applied to estimate the number of ICs in our data. Using principal component analysis, individual data was reduced. Then, the informax algorithm (Bell and Sejnowski, 1995) was applied for the group ICA and estimated 14 components. In order to improve the IC’s stability, the ICASSO was applied and run 20 times (Himberg et al., 2004). Of 14 components, one component related to residual artifact was excluded for further analysis.

Thirteen components were correlated with the brain and defined as brain networks. We labelled them with regional or functional descriptors (e.g., default mode network; DMN, motor network; MN). Then, we examined the experimental condition-relatedness in each network using the temporal sorting in the GIFT. Temporal sorting applied the GLM to the component’s time course. The fMRI specific time course for each individual was regressed against the design matrix for the experimental conditions and tested for significance to identify networks where activity was greater during each condition. The resulting β weights represent the degree to which network was recruited by the conditions. For a network, positive and negative β weights indicate the level of network recruitment in each experimental condition. To assess it, one-sample *t*-tests were conducted on β weights (*p*_FDR-corrected_ < 0.05) in each group (Task M1 TMS group: 13 components, two contrasts, and four conditions; Rest M1 TMS: 13 component and two contrast; Vertex TMS: 13 component and two contrasts). Then, the networks showed the significant recruitment in the experimental conditions were selected for the next analysis. For the task M1 stimulation group, a two-factorial ANOVA with the site of stimulation (left and right) and TMS (TMS and no-TMS) as within-subject factors was performed for the left M1 stimulation and the right M1 stimulation according to each task (LHC, RHC, BHC and rest) separately. For the comparison between TMS and no-TMS phase, planned *t*-tests were performed for each network (*p* < 0.05). In order to evaluate the effect of the M1 stimulation during task and at rest, relative to the control stimulation, one-way ANOVA was conducted across the groups on the resting condition. *Post hoc* tests were performed for each network to compare the effect of TMS site (M1 and vertex; Scheffe’s test, *p* < 0.05).

To assess the connectivity between networks, FNC analysis was performed. The FNC was estimated as the Pearson’s correlation coefficients between pairs of time-courses of networks (Jafri et al., 2008). To explore the FNC changes caused by the group [task M1 (left M1 TMS), rest M1 and vertex stimulation], ANOVA was conducted and following *post hoc* tests were performed (Scheffe’s test, *p* < 0.05).

## Results

### GLM Results

**Figure 2:**
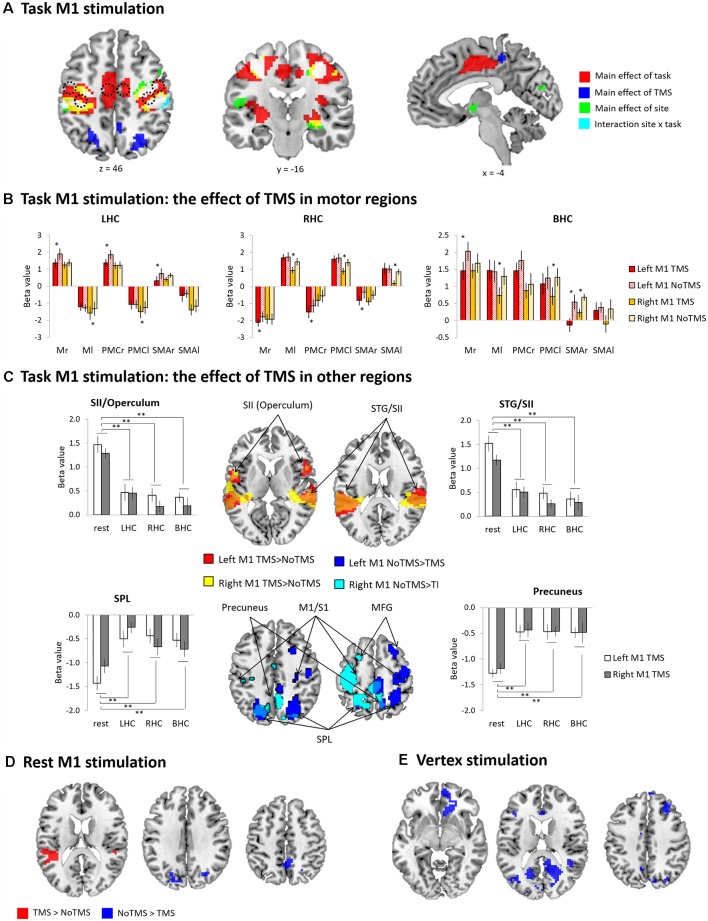
General linear model (GLM) results **(A)** task primary motor cortex (M1) stimulation whole-brain activation map. Red (main effect of task), Blue (main effect of TMS), Green (main effect of site), Cyan (interaction between task and site), and White (overlapping areas across the contrasts). **(B)** The results of ROI analysis in the motor regions [M1, premotor cortex (PMC), and SMA]: Task M1 stimulation. The bar filled color indicates the TMS phase and the bar filled with diagonal lines indicates the No TMS phase. Red (left M1 TMS) and Yellow (right M1 TMS). **(C)** The results of conjunction analysis: Task M1 stimulation. **(D)** Rest M1 stimulation whole-brain activation map. Red (TMS > NoTMS) and Blue (NoTMS > TMS). **(E)** Vertex stimulation whole-brain activation map. Red (TMS > NoTMS) and Blue (NoTMS > TMS). Error bars indicate standard errors. *p < 0.05, **p < 0.005.

The GLM results are summarized in Figure 2A, Supplementary Table S2. The main effect of task revealed that hand movements increased brain activity in the motor system including the bilateral M1, PMC, and SMA as well as the primary sensory cortex (S1), putamen, thalamus and visual cortex. The main effect of TMS was found in the bilateral superior parietal lobe (SPL), precuneus, middle cingulate cortex (MCC) and left superior temporal gyrus (STG). The main effect of TMS site was found in the bilateral M1/S1, inferior frontal gyrus (IFG), left insular, right putamen, STG and middle temporal gyrus (MTG). There was a significant interaction between the site and task in the bilateral M1 and S1. No voxels were survived in the other contrast of interactions.

To investigate the task-specific TMS effects within the MN, repeated-measures ANOVAs with hemisphere (left vs. right), site of stimulation (left vs. right) and TMS (TMS vs. NoTMS) were performed for each ROI (M1, PMC, and SMA) according to task conditions. All results were summarized in Figure 2B and Supplementary Results. During the LHC, we found that the left M1 TMS evoked significant reduction in the regional activity in the right (contralateral) hemisphere, whereas the right M1 TMS induced a significant increase of the magnitude of deactivation in the left motor areas (Figure 2B, LHC). During the RHC, the left M1 TMS evoked the significant increase of deactivations in the contralateral (right) hemisphere, whereas right M1 TMS induced significant decreases of activity in all contralateral ROIs (Figure 2B, RHC). During the BHC, the left M1 TMS evoked a significant reduction in the activity of the contralateral (right) M1 and SMA, whereas the right M1 TMS induced a significant decrease in the activity of the left M1, PMC, as well as the right SMA (Figure 2B, BHC). In the MN, we demonstrated that a short train of 1 Hz TMS to the M1 induced a strong interhemispheric inhibition resulting in decreased activation/increased deactivation in the contralateral regions across the task conditions.

To investigate the M1 TMS effect at the whole brain, we performed a conjunction analysis by comparing TMS with NoTMS phases across the task conditions. Figure 2C summarizes the results. The M1 TMS evoked significant activation in the bilateral secondary somatosensory cortex (SII) including rolandic operculum (RO) and STG as well as deactivation in the contralateral precentral gyrus/postcentral gyrus (M1/S1), middle frontal gyrus (MFG) and bilateral SPL, precuneus, and middle occipital gyrus (MOG). A repeated-measures ANOVA with the task (LHC, RHC, BHC and rest) and TMS site (left vs. right) was performed in these regions. The ANOVAs revealed that a significant effect of task for the all ROIs [SII (operculum): F_(3,9)_ = 33.13, *p* < 0.001; SII (STG): F_(3,9)_ = 26.85, *p* < 0.001; precuneus: F_(3,9)_ = 13.60, *p* < 0.001; SPL: F_(3,9)_ = 11.31, *p* < 0.001]. The other main and interaction effects were not significant (*p*s > 0.2). Subsequent *t*-tests for the overlapping regions demonstrated that the TMS effects were stronger at rest than any other task conditions.

At rest, the left M1 stimulation evoked the significant activation in the bilateral STG/SII and deactivation in the superior occipital gyrus (SOG) and precuneus (Figure 2D, Supplementary Table S2). The vertex stimulation caused significant deactivation in the medial prefrontal cortex (mPFC), superior frontal gyrus (SFG), MFG, precuneus, and visual cortex (Figure 2E, Supplementary Table S2).

### ICA Results

**Figure 3:**
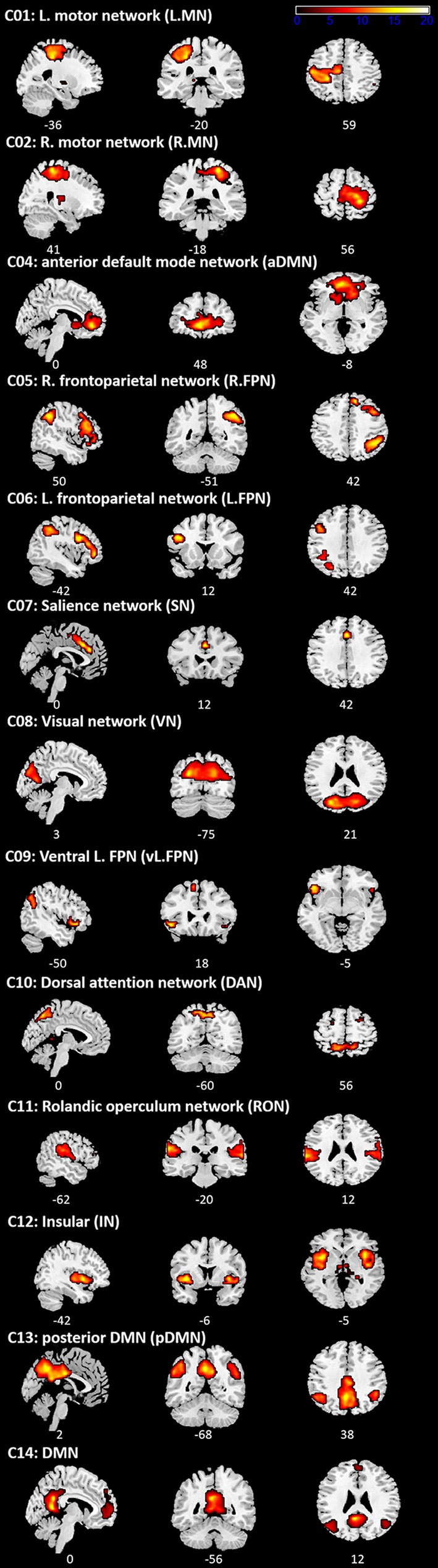
Independent component analysis (ICA) results. Spatial distribution of 13 networks. See Supplementary Table S3 for the Montreal Neurological Institute (MNI) coordinates.

**Figure 4:**
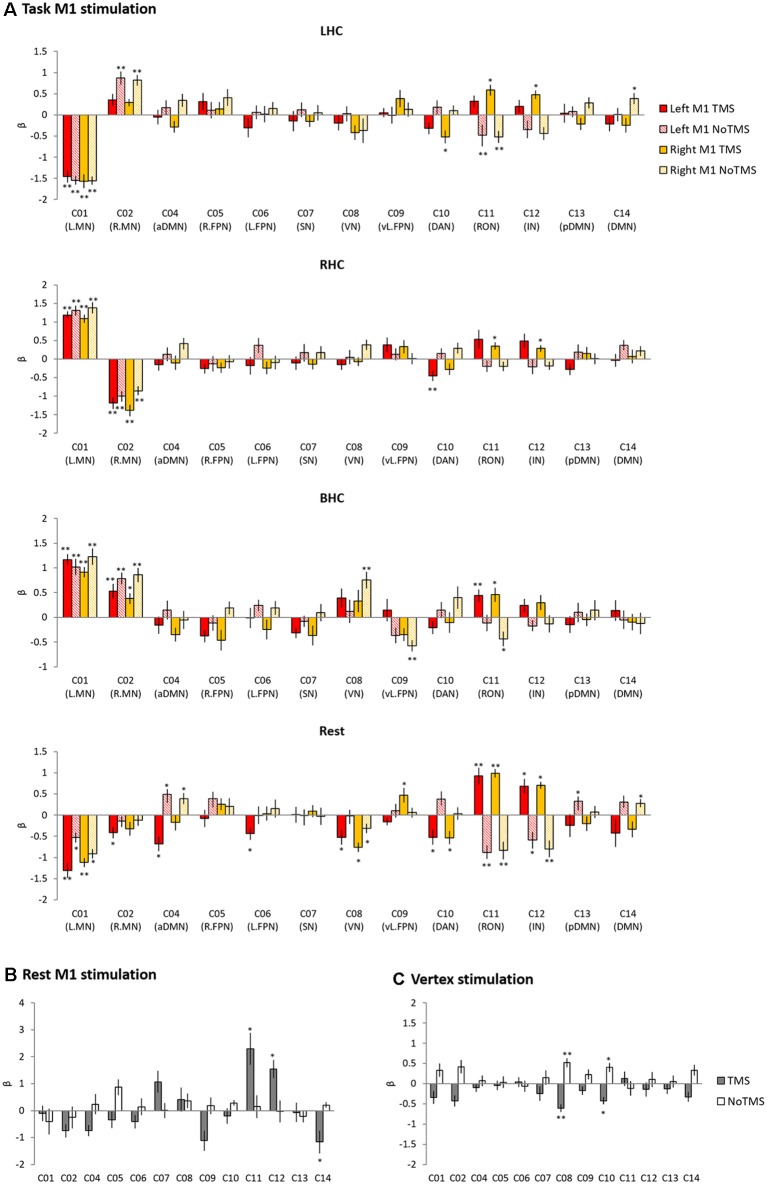
The results of temporal regression analysis. Bar chart showing the mean β value for each condition from a regression analysis performed on each of the 13 networks. **(A)** Task M1 stimulation. **(B)** Rest M1 stimulation. **(C)** Vertex stimulation. Error bars indicate standard errors. *p_FDR-corrected_ < 0.05, **p_FDR-corrected_ < 0.005.

ICA revealed 13 components showing the patterns of temporally coherent signals confined to the brain, which were considered as the functional brain networks (Figure 3, Supplementary Table S3). Figure 4 displays the result of the regression analysis on each network, showing the level of activity of networks by the experimental conditions in each group (task M1, rest M1 and vertex stimulation).

In the task M1 stimulation group (Figure 4A), we found several networks were significantly modulated by the HC task and TMS: C01 (L.MN). C02 (R.MN), C04 (aDMN), C08 (VN), C09 (vl.FPN), C10 (DAN), C11 (RON), and C12 (IN). C01 (L.MN) consisted of the left M1, S1, SMA, RO, insular, putamen and thalamus and C02 (R.MN) included the right M1, S1, SMA, and RO. C04 (aDMN) was composed of the mPFC, anterior cingulate cortex (ACC) and superior medial gyrus (SMG). C08 (VN) consisted of the primary visual cortex. C09 (vl.FPN) included the left IFG, angular gyrus (AG) and inferior parietal lobe (IPL). C10 (DAN) consisted of the bilateral frontal eye field and SPL. C11 (RON) contained the bilateral RO and STG and C12 (IN) was composed of the bilateral insular. The LHC significantly activated C02 (R.MN) without TMS, C11 (RON) and C12 (IN) with the right M1 TMS, whereas deactivated C01 (L.MN), C11 (RON) without TMS and C10 (DAN) with the right M1 TMS. The RHC showed the significant activation in C01 (L.MN) across the all conditions, C11 (RON) and C12 (IN) with the right M1 TMS as well as the deactivation in C02 (R.MN) and C10 (DAN) with the left M1 TMS. The BHC significantly activated C01 (L.MN) and C02 (R.MN) regardless of the conditions. During the BHC, C11 (RON) showed the significant activation with TMS and deactivation without TMS over the right M1. C09 (vl.FPN) was deactivated without TMS over the right M1. The rest condition showed the different patterns of network modulation compared to the task condition. TMS significantly activated C11 (RON) and C12 (IN), whereas deactivated C08 (VN) and C10 (DAN) regardless of the site as well as C02 (R.MN), C04 (aDMN), C06 (L.FPN) with the left M1 TMS. C09 (vl.FPN) showed significant activation with the right M1 TMS.

The rest M1 stimulation group showed that TMS significantly activated C11 (L.MN) and C12 (R.MN) and deactivated C04 (aDMN) and C14 (DMN; Figure 4B). C14 (DMN) consisted of the mPFC, precuneus and bilateral AG. The vertex group showed the deactivation in C08 (VN) and C10 (DAN; Figure 4C).

**Figure 5:**
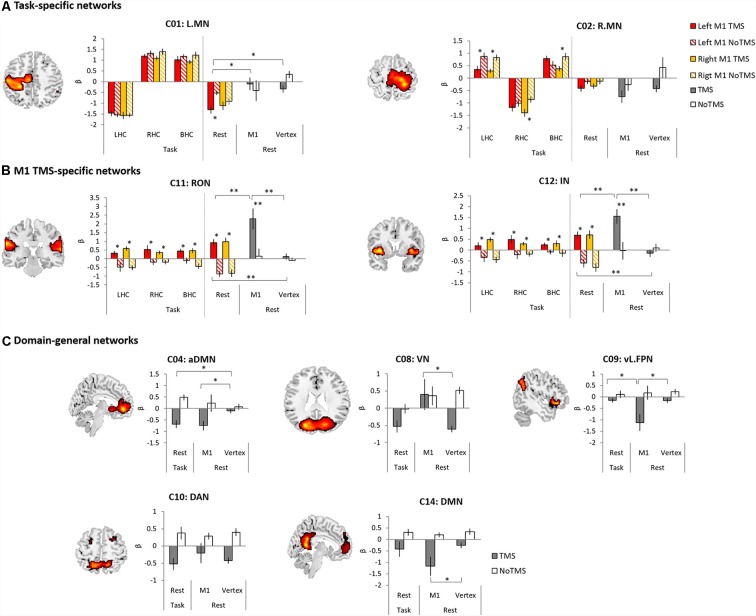
The results of temporal regression analysis between groups. **(A)** Task-specific networks. **(B)** M1 TMS specific networks. **(C)** Domain-general networks. The bar filled color indicates the TMS phase and the bar filled with diagonal lines indicates the No TMS phase. Red (left M1 TMS) and Yellow (right M1 TMS). Gray bar indicated TMS phase and white bar indicate NoTMS phase. Error bars indicate standard errors. *p < 0.05, **p < 0.005.

To investigate the effect of the M1 stimulation at work and rest, we selected the networks significantly modulated by task and TMS (C01, C02, C04, C08, C09, C10, C11, C12 and C14). First, for the task M1 stimulation group, we used 2 × 2 ANOVA with task (LHC, RHC, BHC and rest) and TMS (TMS and no-TMS) as within-subject factors to examine the effect of M1 stimulation during motor execution task. Figure 5A displays the results of the task-specific networks (C01 and C02). C01 (L.MN) showed a significant main effect of task (*F*_(1,11)_ = 62.48, *p* < 0.001) and an interaction (*F*_(3,9)_ = 4.70, *p* = 0.031). *Post hoc* *t*-tests revealed that the left M1 TMS significantly reduced the deactivation at rest. C02 (R.MN) showed the main effect of task (*F*_(1,11)_ = 28.21, *p* < 0.001) and TMS (*F*_(1,11)_ = 4.99, *p* = 0.047). *Post hoc* *t*-tests revealed that the left M1 TMS reduced the activity during the LHC. The right M1 TMS also decreased the activation during the LHC and BHC, whereas increased deactivation during the RHC. Figure 5B shows the result of the M1 TMS-specific networks (C11 and C12). C11 (RON) showed a significant main effect of TMS (*F*_(1,11)_ = 32.11, *p* < 0.001) and an interaction (*F*_(3,9)_ = 6.13, *p* = 0.015). *Post hoc* *t*-tests revealed that the M1 TMS significantly increased the network activity across all task conditions. C12 (IN) showed the significant main effect of TMS (*F*_(1,11)_ = 24.87, *p* < 0.001). *Post hoc* *t*-tests revealed that the M1 TMS significantly increased the network activity across all task conditions.

Second, to compare the effect of the M1 stimulation at rest during task and at rest for the whole fMRI session, ANOVA with TMS (TMS and noTMS) as a within-subject factor and group (task M1, rest M1 and vertex) as a between-subject factor was conducted. The rest condition during task was from the left M1 TMS session. C01 (L.MN) showed the main effect of group (*F*_(2,33)_ = 23.61, *p* < 0.001), whereas C02 (R.MN) showed the main effect of TMS (*F*_(1,33)_ = 5.87, *p* = 0.021) and group (*F*_(2,33)_ = 4.83, *p* = 0.014). *Post hoc* *t*-tests on the TMS phase between groups revealed that the task M1 stimulation group showed the significant deactivation in C01 (L.MN) compared to the rest M1 and vertex (Figure 5A). The M1 TMS-specific networks showed the significant main effect of TMS (C11: *F*_(1,33)_ = 31.22, *p* < 0.001; C12: *F*_(1,33)_ = 15.92, *p* < 0.001) and group (C11: *F*_(2,33)_ = 7.51, *p* = 0.002; C12: *F*_(2,33)_ = 5.62, *p* = 0.008) as well as the interaction (C11: *F*_(2,33)_ = 5.50, *p* = 0.009; C12: *F*_(2,33)_ = 6.67, *p* = 0.004). *Post hoc* *t*-tests on the TMS phase demonstrated that the rest M1 stimulation evoked the stronger networks activation in both C11 (RON) and C12 (IN) compared to other groups and the task M1 stimulation also showed stronger activation in the both networks than vertex group (Figure 5B).

Other domain-general networks were examined to detect the effect of the M1 stimulation (Figure 5C). C04 (aDMN) showed a significant main effect of TMS (*F*_(1,33)_ = 19.68, *p* < 0.001) and a marginally significant interaction (*F*_(2,33)_ = 3.09, *p* = 0.059). *Post hoc* *t*-tests on the TMS phase revealed that both task M1 and rest M1 stimulation significantly decreased the network deactivation relative to the vertex stimulation. C08 (VN) showed a main effect of TMS (*F*_(1,33)_ = 6.81, *p* = 0.014) and group (*F*_(2,33)_ = 4.03, *p* = 0.027). *Post hoc* *t*-tests on the TMS phase revealed that the rest M1 stimulation evoked the increased activation of C08 (VN) relative to the vertex stimulation. C09 (vl.FPN) showed a main effect of TMS (*F*_(1,33)_ = 14.86, *p* = 0.001) and an interaction (*F*_(2,33)_ = 3.69, *p* = 0.036). *Post hoc* *t*-tests on the TMS phase revealed that rest M1 stimulation significantly deactivated the network activity compared to the other groups. We found the main effect of TMS in C10 (DAN; *F*_(1,33)_ = 22.41, *p* < 0.001). C14 (DMN) showed a main effect of TMS (*F*_(1,33)_ = 19.68, *p* = 0.001) and a marginally significant interaction (*F*_(2,33)_ = 2.97, *p* = 0.069). *Post hoc* *t*-tests on the TMS phase revealed that the rest M1 stimulation significantly deactivated the network compared to the vertex stimulation.

**Figure 6:**
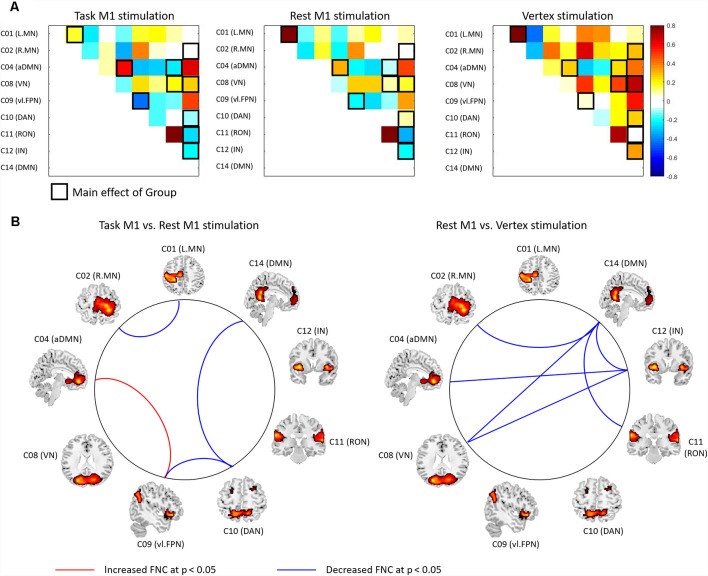
The results of functional network connectivity (FNC) analysis between groups. **(A)** The FNC matrix. Pairwise correlation coefficients between RSN time courses were Fisher z-transformed and averaged across subjects within the task M1 (left M1 TMS session), rest M1 and vertex stimulation groups. The black boxes indicate the FNC that showed a significant group effect (*p* < 0.05). Warm colors indicate the positive coupling and cool colors indicates the negative coupling (decoupling) between the networks. **(B)** The FNC comparisons between two groups (left: Task M1 vs. Rest M1 stimulation; right: rest M1 vs. Vertex stimulation). The red-line indicates the increased FNC and blue line shows the decreased FNC between the groups (*p* < 0.05).

To detect the FNC changes modulated by TMS and task, we conducted a one-way ANOVA with a group [task M1 (left M1 TMS), rest M1 and vertex] on the FNC between the networks showed the effect of them in the previous analysis. We found a significant group effect in the FNC between the networks (C01–C02, C02–C14, C04–C08, C04–C12, C08–C12, C08–C14, C09–C10, C10–C14, C11–C14, and C12–C14; Figure 6A, Supplementary Table S4). *Post hoc* *t*-tests between groups demonstrated that the task M1 group compared to the rest M1 group showed the significantly decreased FNC in C01–C02, C09–C10 and C10–C14 as well as the increased FNC in C04–C09 (Figure 6B, left). The rest M1 stimulation relative to the vertex stimulation showed the decreased FNC in C02–C14, C04–C12, C08–C12, C08–C14 and C11–C14 (Figure 6B, right).

## Discussion

We investigated the acute effect of TMS over the M1 on functional brain networks at work and at rest. Employing the concurrent TMS/fMRI, we assessed the immediate causal changes at the brain activity and network connectivity, reflecting the experience of the M1 stimulation—the intended cortical excitability modulation at the motor cortex and functionally connected regions as well as unintended multisensory perception. This unique experience of the M1 stimulation evoked dynamic changes in within and between functional networks including the targeted motor system and other cognitive networks. Especially, we found selective changes in the networks involved in higher sensory processing and self-related processing. Moreover, the observed neural changes caused by the M1 TMS were more prominent when the neural system was not engaged in a motor task. Our results suggest complex neural dynamics involved in modulating the M1 with TMS and its state-dependency, which has an implication of the underlying neural mechanism of functional reorganization of whole-brain dynamics (Fornito et al., 2012; Cocchi et al., 2015).

A novel finding of this study is that there were two non-motor networks specifically modulated by the M1 stimulation: C11 (RON: rolandic operculum) and C12 (IN: insular). The M1 stimulation evoked activation in these networks regardless of the states. The RO is a part of the SII (Eickhoff et al., 2006) and involved in a wide range of somatic stimuli processing (Ledberg et al., 1995; Roland et al., 1998; Bodegard et al., 2000). Anatomically, this area is directly connected to key multisensory regions including the PMC, M1, IPL, inferior parietal sulcus and inferior frontal cortices and functionally associated with the multisensory perception of the hand through the collaboration with the frontoparietal regions (Eickhoff et al., 2010; Gentile et al., 2011). TMS over the M1 induces peripheral muscle activity at the targeted hand. A previous study has shown that this afferent feedback caused by TMS contributed to the BOLD signal changes in the M1 and somatosensory areas (Denslow et al., 2005). However, the M1 TMS generates not only physical muscle responses but also the awareness of the unintended muscle activity. Participants experienced that their hand was strained or sometimes twitched by the stimulation, not by themselves, which could alert the sense of ownership or agency for one’s hand and its movements (Murray and Wallace, 2012). Thus, this unique experience of the M1 stimulation can drive bodily self-consciousness (BSC)—the processing and integration of multisensory bodily signals (Lenggenhager et al., 2007; Blanke, 2012). The RO has been repeatedly associated with the BSC, showing increased activation in interoceptive signals from the body parts (Damasio and Meyer, 2009; Blefari et al., 2017). In addition to the RO, the insular play a critical role in BSC (Damasio and Meyer, 2009; Blanke, 2012; Gogolla, 2017). The insular is located deep within the lateral sulcus of each hemisphere, heavily connected to cortical and subcortical regions serving sensory, emotion and cognitive functions (Shelley and Trimble, 2004). The insular receives information from outside the body (auditory, somatosensory, olfactory, gustatory and visual information) and from inside the body (interoceptive information). Based on anatomical and functional connectivity of the insular, Craig suggested that the insula is a locus to form the self-awareness of feelings from the body (Craig, 2011). Thus, the strong regional activity and the increased network activation in the RO and insular may be attributed to not only the afferent/reafferent feedback but also the self-awareness of feeling caused by the multisensory experience from the M1 TMS.

These findings have important implications for understanding the mechanism underpinning large-scale neural dynamics. Cocchi et al. (2015) delivered repetitive TMS between two resting-state fMRI sessions (before and after stimulation sessions) and applied graph-theory network analysis to investigate the impact of local changes on intrinsic whole-brain dynamics. They found that after the continuous theta-burst stimulation (cTBS) over the right motor cortex, the sensorimotor system and insular became more integrated but no changes overall pattern of large-scale integration. They suggested that a local change of neural activity (cTBS modulation of the M1) was progressively integrated into whole-brain dynamics by hierarchical mechanisms, balancing between local neural specialization and global integration (Fornito et al., 2012; van den Heuvel and Sporns, 2013). Consistently, our results showed selective neural changes in the targeted motor system and higher sensory systems. Specifically, the M1 TMS decreased the regional activity and within network connectivity in the motor regions, whereas increased them in the RO and insular. In addition, the observed network-level changes were state-dependent: stronger activation at rest and even in the rest condition during motor execution (Bestmann et al., 2008; Silvanto and Pascual-Leone, 2008). Furthermore, these networks were decoupled with the DMN when the TMS was applied over the M1 compared to the vertex. The DMN shows task-related deactivation and involved in internally focused processing such as mind-wandering and consciousness (Buckner et al., 2008), consisting of the key regions of the integrative hubs including the mPFC and precuneus (van den Heuvel and Sporns, 2013). Thus, our results suggests that the M1 stimulation prompts acute local changes at the selective networks by dissociating them from the higher systems associated with global integration and the underlying neural mechanism of brain dynamics may depend on specific network states (rest vs. task).

In the motor system, we found that a short train of 1 Hz TMS to the M1 induced the strong interhemispheric inhibition during motor execution at the regional activity as well as the FNC between the left and right motor networks. The interhemispheric inhibition caused by TMS has been widely investigated in both humans and animals (Chang, 1953; Matsunami and Hamada, 1984; Ferbert et al., 1992; Wassermann et al., 1998; Hanajima et al., 2001) suggesting that the interhemispheric inhibition is mediated through transcallosal pathways (Meyer et al., 1995, 1998). Recently, studies combining TMS with neuroimaging (PET/fMRI) have showed that TMS over the M1 modulated brain activity in the motor areas connected to the targeted M1 (Bohning et al., 1999, 2000a,b; Bestmann et al., 2003, 2004). In accordance with previous studies, our ROI results demonstrated that the M1 stimulation induced the interhemispheric inhibition, decreasing the task-related activation and increasing the deactivation at the contralateral motor regions. However, at the network level, we found that the TMS effect in the MN of the dominant hemisphere (left) was not evident as that in the non-dominant MN (R.MN). R.MN showed the significant TMS effect, showing the reduction of network activation and the enlargement of network deactivation. Contrary to R.MN, M1 TMS did not modulate L.MN during the task but the left M1 TMS at rest increased the network deactivation. It might be possible that the current TMS paradigm (11 pulses with 100% RMT) was not enough to elicit neural changes at the network level when the network linked to the dominant hand. This finding is compatible with previous reports demonstrating the absence of significant alterations in brain activity at the M1 after TMS (Bohning et al., 2000a; Baudewig et al., 2001). Bohning et al. (2000a) delivered 1 Hz TMS with 110% RMT over the M1 and estimated the level of regional activity at the target region during a simple motor execution task. The regional activity associated with the task and TMS was not different from the activity evoked by the task alone.

We showed that the M1 stimulation deactivated other domain-general networks. Previously, we demonstrated that the M1 stimulation inhibited the DMN compared to the vertex stimulation (Jung et al., 2016). Similarly, we found that the M1 stimulation deactivated aDMN (C04) and DMN (C14) relative to the vertex stimulation. The experience of the M1 TMS disrupts the internally focused processing, leading to the deactivation of the DMN (Jung et al., 2016). A task-active network (C09) showed a state-dependent TMS effect. The vl.FPN (C09) is a sub-set of the FPN contributing to executive processing across tasks (Fedorenko et al., 2013). It might be driven from the uncontrolled mental activity at rest, not directly connected to the M1 stimulation. The DAN (C10) showed the deactivation when TMS was delivered to the M1 or vertex. The DAN is involved in the top-down guided voluntary allocation of attention to sensory input (Vossel et al., 2014). The general by-product of TMS pulses such as tactile sensation and “click” sound potentially contributed to the deactivation of the network. In addition to the TMS effect, the task also modulated the interaction between these networks—decoupling task-active network (DAN) and task-negative network (DMN).

We found that the vertex stimulation reduced the network activity of the VN (C08) and DAN (C10). During the vertex TMS, participants were instructed to see the fixation on the screen. The increased activity of the VN and DAN could be associated with visual processing without the stimulation. The vertex stimulation might disrupt the ongoing visual and attention processing due to the by-product of TMS (Wasserman et al., 2008; Jung et al., 2016).

The current study has several limitations. First, the sample size of the study is relatively small and 75% of participants were female. There is emerging evidence that gender can be a factor to determine the TMS-induced plasticity (Ridding and Ziemann, 2010). In order to test the gender effect, we re-analyzed the data by adding the gender as an additional factor and found no significant effect of the gender in our key findings. Second, the vertex stimulation group received 120% of the RMT, which was stronger than the other groups (100% RMT). The intensity of TMS is another factor contributing to the TMS effects: the stronger intensity, the bigger TMS effects (Wasserman et al., 2008). However, we found no significant effect of the vertex stimulation on the key findings in this study (no effect at the target site and key networks including the MN, RON, IN, and DMN). Thus, the vertex stimulation performed its role as a control site (Jung et al., 2016). Future studies will be needed with a larger sample size by controlling other confounding factors for the TMS effect to elucidate the neural mechanism of the M1 stimulation.

## Data Availability Statement

The datasets generated for this study are available on request to the corresponding author.

## Ethics Statement

The studies involving human participants were reviewed and approved by Korea University. The patients/participants provided their written informed consent to participate in this study.

## Author Contributions

JJ and SJ designed the study, wrote the manuscript and approved the final manuscript. AB and RB developed the concurrent TMS/fMRI system. JJ and AB collected the data. JJ analyzed the data.

## Conflict of Interest

The authors declare that the research was conducted in the absence of any commercial or financial relationships that could be construed as a potential conflict of interest.

## Abbreviations

M1 TMS: TMS over the primary motor cortex
HC: hand clenching
MN: motor network
DMN: default mode network
FPN: frontoparietal network
VN: visual network
DAN: dorsal attention network
RO: rolandic operculum
RON: rolandic operculum network
IN: insular network
FNC: functional network connectivity
BSC: bodily self-consciousness.

## References

[B1] Baudewig J. Siebner H. R. Bestmann S. Tergau F. Tings T. Paulus W.. (2001). Functional MRI of cortical activations induced by transcranial magnetic stimulation (TMS). Neuroreport 12, 3543–3548. 10.1097/00001756-200111160-00034 11733708

[B2] Bell A. J. Sejnowski T. J. (1995). An information-maximization approach to blind separation and blind deconvolution. Neural Comput. 7, 1129–1159. 10.1162/neco.1995.7.6.1129 7584893

[B3] Bestmann S. Baudewig J. Siebner H. R. Rothwell J. C. Frahm J. (2003). Subthreshold high-frequency TMS of human primary motor cortex modulates interconnected frontal motor areas as detected by interleaved fMRI-TMS. NeuroImage 20, 1685–1696. 10.1016/j.neuroimage.2003.07.028 14642478

[B4] Bestmann S. Baudewig J. Siebner H. R. Rothwell J. C. Frahm J. (2004). Functional MRI of the immediate impact of transcranial magnetic stimulation on cortical and subcortical motor circuits. Eur. J. Neurosci. 19, 1950–1962. 10.1111/j.1460-9568.2004.03277.x 15078569

[B5] Bestmann S. Baudewig J. Siebner H. R. Rothwell J. C. Frahm J. (2005). BOLD MRI responses to repetitive TMS over human dorsal premotor cortex. NeuroImage 28, 22–29. 10.1016/j.neuroimage.2005.05.027 16002305

[B6] Bestmann S. Swayne O. Blankenburg F. Ruff C. C. Haggard P. Weiskopf N.. (2008). Dorsal premotor cortex exerts state-dependent causal influences on activity in contralateral primary motor and dorsal premotor cortex. Cereb. Cortex 18, 1281–1291. 10.1093/cercor/bhm159 17965128 PMC2600427

[B7] Blanke O. (2012). Multisensory brain mechanisms of bodily self-consciousness. Nat. Rev. Neurosci. 13, 556–571. 10.1038/nrn3292 22805909

[B8] Blefari M. L. Martuzzi R. Salomon R. Bello-Ruiz J. Herbelin B. Serino A.. (2017). Bilateral Rolandic operculum processing underlying heartbeat awareness reflects changes in bodily self-consciousness. Eur. J. Neurosci. 45, 1300–1312. 10.1111/ejn.13567 28370498

[B9] Bodegard A. Ledberg A. Geyer S. Naito E. Zilles K. Roland P. E. (2000). Object shape differences reflected by somatosensory cortical activation. J. Neurosci. 20:RC51. 10.1523/JNEUROSCI.20-01-j0004.2000 10627628 PMC6774143

[B10] Bohning D. Shastri A. Lomarev M. Lorberbaum J. P. Nahas Z. George M. (2003). BOLD-fMRI vs. transcranial magnetic stimulation (TMS) pulse-train length: testing for linearity. J. Magn. Reson. Imaging 17, 279–290. 10.1002/jmri.10271 12594717

[B11] Bohning D. Shastri A. McConnell K. Nahas Z. Lorberbaum J. Robert D.. (1999). A combined TMS/fMRI study of intensity-dependent TMS over motor cortex. Biol. Psychiatry 45, 385–394. 10.1016/s0006-3223(98)00368-0 10071706

[B12] Bohning D. Shastri A. McGavin L. McConnell K. Nahas Z. Lorberbaum J.. (2000a). Motor cortex brain activity induced by 1-Hz transcanial magnetic stimulation is stimilar in location and level to that for volitional movement. Invest. Radiol. 35, 676–683. 10.1097/00004424-200011000-00005 11110304

[B14] Bohning D. Shastri A. Wassermann E. Ziemann U. Lorberbaum J. Nahas Z.. (2000b). BOLD-fMRI response to single-pulse transcanial magnetic stimulation (TMS). J. Magn. Reson. Imaging 11, 569–574. 10.1002/1522-2586(200006)11:6<569::aid-jmri1>3.0.co;2-3 10862054

[B13] Bohning D. Shastri A. Nahas Z. Lorberbaum J. Andersen S. Danels W.. (1998). Echo-planar BOLD fMRI of brain acitvation induced by concurrent transcanial magnetic stimulation. Invest. Radiol. 33, 336–340. 10.1097/00004424-199806000-00004 9647445

[B15] Buckner R. L. Andrews-Hanna J. R. Schacter D. L. (2008). The brain’s default network: anatomy, function, and relevance to disease. Ann. N Y Acad. Sci. 1124, 1–38. 10.1196/annals.1440.011 18400922

[B16] Calhoun V. D. Adali T. Pearlson G. D. Pekar J. J. (2001). A method for making group inferences from functional MRI data using independent component analysis. Hum. Brain Mapp. 14, 140–151. 10.1002/hbm.1048 11559959 PMC6871952

[B17] Chang H. T. (1953). Cortical response to activity of callosal neurons. J. Neurophysiol. 16, 117–131. 10.1152/jn.1953.16.2.117 13035471

[B18] Cocchi L. Sale M. V. Lord A. Zalesky A. Breakspear M. Mattingley J. B. (2015). Dissociable effects of local inhibitory and excitatory theta-burst stimulation on large-scale brain dynamics. J. Neurophysiol. 113, 3375–3385. 10.1152/jn.00850.2014 25717162 PMC4443609

[B19] Craig A. D. (2011). Significance of the insula for the evolution of human awareness of feelings from the body. Ann. N Y Acad. Sci. 1225, 72–82. 10.1111/j.1749-6632.2011.05990.x 21534994

[B20] Damasio A. Meyer K. (2009). “Consciousness: an overview of the phenomenon and of its possible neural basis,” in Neurology of Consciousness: Cognitive Neuroscience and Neuropathology, eds Laureys S. Tononi G. (Oxford, UK: Academic Press), 3–14.

[B21] Denslow S. Lomarev M. George M. S. Bohning D. E. (2005). Cortical and subcortical brain effects of transcranial magnetic stimulation (TMS)-induced movement: an interleaved TMS/functional magnetic resonance imaging study. Biol. Psychiatry 57, 752–760. 10.1016/j.biopsych.2004.12.017 15820232

[B22] Di Lazzaro V. Oliviero A. Profice P. Insola A. Mazzone P. Tonali P. (1999). Direct demonstration of interhemispheric inhibition of the human motor cortex produced by transcranial magnetic stimulation. Exp. Brain Res. 124, 520–524. 10.1007/s002210050648 10090664

[B23] Egolf E. Kiehl K. A. Calhoun V. D. (2004). Group ICA of fMRI toolbox (GIFT). Budapest, Hungary: Proc HBM.

[B24] Eickhoff S. B. Jbabdi S. Caspers S. Laird A. R. Fox P. T. Zilles K.. (2010). Anatomical and functional connectivity of cytoarchitectonic areas within the human parietal operculum. J. Neurosci. 30, 6409–6421. 10.1523/JNEUROSCI.5664-09.2010 20445067 PMC4791040

[B25] Eickhoff S. B. Schleicher A. Zilles K. Amunts K. (2006). The human parietal operculum: I. Cytoarchitectonic mapping of subdivisions. Cereb. Cortex 16, 254–267. 10.1093/cercor/bhi105 15888607

[B26] Fedorenko E. Duncan J. Kanwisher N. (2013). Broad domain generality in focal regions of frontal and parietal cortex. Proc. Natl. Acad. Sci. U S A 110, 16616–16621. 10.1073/pnas.1315235110 24062451 PMC3799302

[B27] Ferbert A. Priori A. Rothwell J. C. Day B. L. Colebatch J. G. Marsden C. D. (1992). Interhemispheric inhibition of the human motor cortex. J. Physiol. 453, 525–546. 10.1113/jphysiol.1992.sp019243 1464843 PMC1175572

[B28] Fornito A. Harrison B. J. Zalesky A. Simons J. S. (2012). Competitive and cooperative dynamics of large-scale brain functional networks supporting recollection. Proc. Natl. Acad. Sci. U S A 109, 12788–12793. 10.1073/pnas.1204185109 22807481 PMC3412011

[B29] Gentile G. Petkova V. I. Ehrsson H. H. (2011). Integration of visual and tactile signals from the hand in the human brain: an FMRI study. J. Neurophysiol. 105, 910–922. 10.1152/jn.00840.2010 21148091 PMC3059180

[B30] Gogolla N. (2017). The insular cortex. Curr. Biol. 27, R580–R586. 10.1016/j.cub.2017.05.010 28633023

[B31] Hanajima R. Ugawa Y. Machii K. Mochizuki H. Terao Y. Enomoto H.. (2001). Interhemispheric facilitation of the hand motor area in humans. J. Physiol. 531, 849–859. 10.1111/j.1469-7793.2001.0849h.x 11251064 PMC2278503

[B32] Himberg J. Hyvärinen A. Esposito F. (2004). Validating the independent components of neuroimaging time series *via* clustering and visualization. NeuroImage 22, 1214–1222. 10.1016/s1053-8119(04)00166-1 15219593

[B33] Jafri M. J. Pearlson G. D. Stevens M. Calhoun V. D. (2008). A method for functional network connectivity among spatially independent resting-state components in schizophrenia. NeuroImage 39, 1666–1681. 10.1016/j.neuroimage.2007.11.001 18082428 PMC3164840

[B34] Jung J. Bungert A. Bowtell R. Jackson S. R. (2016). Vertex stimulation as a control site for transcranial magnetic stimulation: a concurrent TMS/fMRI study. Brain Stimul. 9, 58–64. 10.1016/j.brs.2015.09.008 26508284 PMC4720218

[B35] Koch G. Franca M. Albrecht U.-V. Caltagirone C. Rothwell J. (2006). Effects of paired pulse tms of primary somatosensory cortex on perception of a peripheral electrical stimulus. Exp. Brain Res. 172, 416–424. 10.1007/s00221-006-0359-0 16523332

[B36] Ledberg A. O’Sullivan B. T. Kinomura S. Roland P. E. (1995). Somatosensory activations of the parietal operculum of man. A PET study. Eur. J. Neurosci. 7, 1934–1941. 10.1111/j.1460-9568.1995.tb00716.x 8528469

[B37] Lenggenhager B. Tadi T. Metzinger T. Blanke O. (2007). Video ergo sum: manipulating bodily self-consciousness. Science 317, 1096–1099. 10.1126/science.1143439 17717189

[B38] Li X. Tenebäck C. C. Nahas Z. Kozel F. A. Large C. Cohn J.. (2004). Interleaved transcranial magnetic stimulation/functional MRI confirms that lamotrigine inhibits cortical excitability in healthy young men. Neuropsychopharmacology 29, 1395–1407. 10.1038/sj.npp.1300452 15100699

[B39] Matsunami K. Hamada I. (1984). Effects of stimulation of corpus callosum on precentral neuron activity in the awake monkey. J. Neurophysiol. 52, 676–691. 10.1152/jn.1984.52.4.676 6491712

[B41] Meyer B. U. Röricht S. Gräfin Von Einsiedel H. Kruggel F. Weindl A. (1995). Inhibitory and excitatory interhemispheric transfers between motor cortical areas in normal humans and patients with abnormalities of the corpus callosum. Brain 118, 429–440. 10.1093/brain/118.2.429 7735884

[B40] Meyer B. U. Röricht S. Woiciechowsky C. (1998). Topography of fibers in the human corpus callosum mediating interhemispheric inhibition between the motor cortices. Ann. Neurol. 43, 360–369. 10.1002/ana.410430314 9506553

[B42] Murray M. M. Wallace M. T. (2012). The Neural Bases of Multisensory Processes. Boca Raton, FL: CRC Press. 22593873

[B43] Oldfield R. C. (1971). The assessment and analysis of handedness: the Edinburgh inventory. Neuropsychologia 9, 97–113. 10.1016/0028-3932(71)90067-4 5146491

[B44] Ridding M. C. Ziemann U. (2010). Determinants of the induction of cortical plasticity by non-invasive brain stimulation in healthy subjects. J. Physiol. 588, 2291–2304. 10.1113/jphysiol.2010.190314 20478978 PMC2915507

[B45] Roland P. E. O’Sullivan B. Kawashima R. (1998). Shape and roughness activate different somatosensory areas in the human brain. Proc. Natl. Acad. Sci. U S A 95, 3295–3300. 10.1073/pnas.95.6.3295 9501256 PMC19735

[B46] Ruff C. C. Blankenburg F. Bjoertomt O. Bestmann S. Weiskopf N. Driver J. (2009). Hemispheric differences in frontal and parietal influences on human occipital cortex: direct confirmation with concurrent TMS-fMRI. J. Cogn. Neurosci. 21, 1146–1161. 10.1162/jocn.2009.21097 18752395 PMC2667814

[B47] Shastri A. George M. S. Bohning D. E. (1999). Performance of a system for interleaving transcranial magnetic stimulation with steady-state magnetic resonance imaging. Electroencephalogr. Clin. Neurophysiol. Suppl. 51, 55–64. 10590936

[B48] Shelley B. P. Trimble M. R. (2004). The insular lobe of Reil—its anatamico-functional, behavioural and neuropsychiatric attributes in humans—a review. World J. Biol. Psychiatry 5, 176–200. 10.1080/15622970410029933 15543513

[B49] Silvanto J. Pascual-Leone A. (2008). State-dependency of transcranial magnetic stimulation. Brain Topogr. 21, 1–10. 10.1007/s10548-008-0067-0 18791818 PMC3049188

[B50] Steinmetz H. Fürst G. Meyer B. U. (1989). Craniocerebral topography within the international 10-20 system. Electroencephalogr. Clin. Neurophysiol. 72, 499–506. 10.1016/0013-4694(89)90227-7 2471619

[B51] van den Heuvel M. P. Sporns O. (2013). Network hubs in the human brain. Trends Cogn. Sci. 17, 683–696. 10.1016/j.tics.2013.09.012 24231140

[B52] Vossel S. Geng J. J. Fink G. R. (2014). Dorsal and ventral attention systems: distinct neural circuits but collaborative roles. Neuroscientist 20, 150–159. 10.1177/1073858413494269 23835449 PMC4107817

[B53] Walsh V. Cowey A. (2000). Transcaniall magnetic stimulation and cognitive neuroscience. Nat. Rev. Neurosci. 1, 73–80. 10.1038/35036239 11252771

[B54] Wasserman E. M. Epstein C. M. Ziemann U. (2008). The Oxford Handbook of Transcranial Stimulation. Oxford; New York, NY: Oxford University Press.

[B55] Wassermann E. M. Wedegaertner F. R. Ziemann U. George M. S. Chen R. (1998). Crossed reduction of human motor cortex excitability by 1-Hz transcranial magnetic stimulation. Neurosci. Lett. 250, 141–144. 10.1016/s0304-3940(98)00437-6 9708852

