# Supplementary material for: Modulating Brain Networks With Transcranial Magnetic Stimulation Over the Primary Motor Cortex: A Concurrent TMS/fMRI Study
Source: Front Hum Neurosci. 2020 Feb 14;14:31. doi: 10.3389/fnhum.2020.00031 (PMC7033446; doi:10.3389/fnhum.2020.00031)
Supplement: Supplementary file 1 [file Table_1.DOCX]

**Supplementary Information**

- Supplementary Results
- Supplementary Table 1-4

**Supplementary Results**

During LHC, there was a significant main effect of hemisphere in all ROIs (M1: F1, 11 = 226.98, p < 0.001; PMC: F1, 11 = 160.22, p < 0.001; SMA: F1, 11 = 53.58, p < 0.001) and a significant TMS effect in the SMA (F1, 11 = 6.50, p < 0.05). The M1 (F1,11 = 3.82, p = 0.08) and the PMC (F1, 11 = 3.84, p = 0.08) showed a marginally significant TMS effect. There was a significant interaction between hemisphere × TMS in the M1 (F1, 11 = 19.96, p < 0.001) and between hemisphere × site × TMS in the M1 (F1, 11 = 10.79, p < 0.01) and the PMC (F1, 11 = 15.10, p < 0.01). Post-hoc comparisons between TMS and NoTMS phases showed that the left M1 TMS evoked significant reduction in the regional activity in the right (contralateral) hemisphere, whereas the right M1 TMS induced a significant increase of the magnitude of deactivation in the left motor areas. There was no TMS effect in the ROIs at the stimulated (ipsilateral) hemisphere. During RHC, the motor regions showed the similar pattern of activity to the LHC condition. There was a significant main effects of hemisphere (M1: F1, 11 = 176.48, p < 0.001; PMC: F1, 11 = 81.43, p < 0.001; SMA: F1, 11 = 48.80, p < 0.001) and TMS (M1: F1, 11 = 5.64, p < 0.05; PMC: F1, 11 = 15.11, p < 0.01; SMA: F1, 11 = 11.11, p < 0.01). The 3 way interaction of hemisphere × site × TMS was significant for all ROIs (M1: F1, 11 = 14.06, p < 0.01; PMC: F1, 11 = 23.50, p < 0.001; SMA: F1, 11 = 8.58, p < 0.05). Post-hoc comparisons between TMS and NoTMS phase revealed the similar findings to the results of the LHC: the left M1 TMS evoked the significant increase of deactivations in the contralateral (right) hemisphere, whereas right M1 TMS induced significant decreases of activity in all contralateral ROIs. During BHC, the ANOVAs revealed a significant main effect of TMS (M1: F1, 11 = 11.51, p < 0.01; PMC: F1, 11 = 6.67, p < 0.05; SMA: F1, 11 = 10.48, p < 0.01). Only the M1 showed a significant main effect of hemisphere (M1: F1, 11 = 7.92, p < 0.001). There was a significant interaction between hemisphere × site × TMS in the M1 (F1, 11 = 12.77, p < 0.01) and SMA (F1, 11 = 4.75, p = 0.05). Post-hoc comparisons between TMS and NoTMS phases showed that the left M1 TMS evoked a significant reduction in the activity of the contralateral (right) M1 and SMA, whereas the right M1 TMS induced a significant decrease in the activity of the left M1, PMC, as well as the right SMA.

**Supplementary Table 1**

| Task M1 stimulation | | | | | | |
| --- | --- | --- | --- | --- | --- | --- |
|  | x | y | z | x | y | z |
| Sub01 | -40 | -12 | 66 | 34 | -8 | 68 |
| Sub02 | -21 | -30 | 74 | 16 | -14 | 78 |
| Sub03 | -34 | -18 | 70 | 34 | -4 | 68 |
| Sub04 | -36 | -24 | 70 | 36 | -2 | 66 |
| Sub05 | -36 | -4 | 66 | 35 | -10 | 60 |
| Sub06 | 40 | -26 | 68 | 30 | 0 | 70 |
| Sub07 | -30 | -42 | 74 | 38 | -18 | 68 |
| Sub08 | -38 | -16 | 68 | 22 | -12 | 74 |
| Sub09 | -40 | -22 | 68 | 36 | -10 | 68 |
| Sub10 | -36 | -18 | 70 | 36 | -14 | 68 |
| Sub11 | -30 | -24 | 72 | 32 | -24 | 74 |
| Sub12 | -30 | -2 | 70 | 30 | -4 | 68 |
| Rest M1 stimulation | | | | | | |
| Sub01 | -34 | -26 | 72 |  |  |  |
| Sub02 | -52 | -26 | 60 |  |  |  |
| Sub03 | -26 | -32 | 76 |  |  |  |
| Sub04 | -34 | -4 | 68 |  |  |  |
| Sub05 | -38 | 16 | 60 |  |  |  |
| Sub06 | -24 | -54 | 74 |  |  |  |
| Sub07 | -36 | -26 | 72 |  |  |  |
| Sub08 | -30 | -38 | 72 |  |  |  |
| Sub09 | -28 | -30 | 74 |  |  |  |
| Sub10 | -36 | -4 | 66 |  |  |  |
| Sub11 | -36 | -24 | 70 |  |  |  |
| Sub12 | -38 | -16 | 68 |  |  |  |
| Vertex stimulation | | | | | | |
| Sub01 | 6 | -12 | 76 |  |  |  |
| Sub02 | 0 | -44 | 72 |  |  |  |
| Sub03 | -4 | -52 | 72 |  |  |  |
| Sub04 | 2 | -10 | 70 |  |  |  |
| Sub05 | 3 | -15 | 72 |  |  |  |
| Sub06 | -2 | -20 | 73 |  |  |  |
| Sub07 | 5 | -16 | 74 |  |  |  |
| Sub08 | -5 | -13 | 75 |  |  |  |
| Sub09 | 0 | -15 | 70 |  |  |  |
| Sub10 | -3 | 5 | 75 |  |  |  |
| Sub11 | 1 | 2 | 76 |  |  |  |
| Sub12 | -1 | 1 | 73 |  |  |  |

Table S1. The MNI coordinates of TMS target site

**Supplementary Table 2**

| Group | Contrasts | Cluster region | Cluster extent | Peak MNI coordinate | | |
| --- | --- | --- | --- | --- | --- | --- |
|  |  |  |  | x | y | z |
| Task M1 stimulation | Main effect of task | M1 | 1273 | -33 | -24 | 63 |
|  |  | S1 |  | -36 | -24 | 51 |
|  |  | PMC |  | -50 | -15 | 37 |
|  |  | SMA |  | -3 | -6 | 48 |
|  |  | M1 | 770 | 36 | -15 | 54 |
|  |  | PMC |  | 52 | -12 | 38 |
|  |  | SMA |  | 6 | -6 | 48 |
|  |  | Thalamus | 450 | -15 | -27 | 0 |
|  |  | Putamen |  | -30 | -15 | -3 |
|  |  | RO |  | -39 | -21 | 18 |
|  |  | Thalamus | 356 | 15 | -24 | 0 |
|  |  | Pallidum |  | 30 | -9 | -6 |
|  |  | RO |  | 39 | -18 | 15 |
|  |  | MOG | 88 | -18 | -90 | 15 |
|  |  | SOG |  | -15 | -81 | 30 |
|  |  | MCC | 69 | 12 | -6 | 45 |
|  |  | Calcarine gyrus | 69 | 15 | -84 | 9 |
|  | Main effect of site | Calcarine gyrus | 354 | 18 | -51 | 12 |
|  |  | STG |  | 51 | -45 | 12 |
|  |  | MTG |  | 66 | -45 | 6 |
|  |  | Lingual gyrus |  | 18 | -54 | 3 |
|  |  | S1 | 325 | -36 | -30 | 54 |
|  |  | IPL |  | -45 | -27 | 45 |
|  |  | SPL |  | -33 | -45 | 57 |
|  |  | IFG | 324 | 51 | 30 | -6 |
|  |  | Putamen |  | 27 | 15 | 9 |
|  |  | M1 | 202 | 36 | -12 | 51 |
|  |  | S1 | 179 | 42 | -30 | 57 |
|  |  | M1 | 125 | -36 | -9 | 48 |
|  |  | Calcarine gyrus | 109 | -15 | -78 | 15 |
|  |  | Cuneus |  | 3 | -81 | 18 |
|  |  | Insular | 54 | -30 | 27 | 9 |
|  |  | RO | 52 | -51 | -18 | 15 |
|  |  | IFG | 47 | -36 | 6 | 24 |
|  |  | SMG | 46 | 6 | 63 | 18 |
|  | Main effect of TMS | SPL | 229 | 24 | -72 | 48 |
|  |  | MOG |  | 33 | -69 | 33 |
|  |  | Precuneus |  | 12 | -57 | 60 |
|  |  | Supramarginal gyrus | 116 | -45 | -36 | 24 |
|  |  | STG |  | -57 | -45 | 15 |
|  |  | Precuneus | 96 | -9 | -57 | 60 |
|  |  | SPL |  | -21 | -54 | 54 |
|  |  | MCC | 34 | 0 | -36 | 51 |
|  | Interaction Task x Site | S1 | 155 | 48 | -27 | 48 |
|  |  | S1 | 123 | -36 | -30 | 54 |
|  |  | IPL |  | -57 | -27 | 45 |
|  |  | M1 | 122 | 36 | -15 | 51 |
|  |  | SFG |  | 27 | -9 | 66 |
|  |  | M1 | 91 | -33 | -15 | 54 |
| Rest M1 stimulation | TMS > NoTMS | STG/SII | 76 | -39 | -39 | 18 |
|  | NoTMS > TMS | SOG | 48 | -21 | -72 | 39 |
|  |  | Precuenus | 31 | 3 | -48 | 48 |
|  |  | SOG | 30 | 27 | -69 | 30 |
| Vertex stimulation | TMS > NoTMS | ─ | ─ | ─ | ─ | ─ |
|  | NoTMS > TMS | MOG | 768 | -30 | -84 | 24 |
|  |  | SOG |  | 30 | -75 | 42 |
|  |  | Precuenus |  | 39 | -66 | 27 |
|  |  | SFG | 157 | -21 | 0 | 48 |
|  |  | MFG |  | -30 | 6 | 57 |
|  |  | mPFC | 107 | 9 | 54 | -9 |
|  |  | MFG | 102 | 27 | 27 | 39 |
|  |  | Precuenus | 21 | 0 | -63 | 51 |

Table S2. The results of GLM analysis

**Supplementary Table 3**

| Component | Cluster region | Cluster extent | Peak MNI coordinate | | |
| --- | --- | --- | --- | --- | --- |
|  |  |  | x | y | z |
| C01 (L.MN) | M1 | 1843 | -30 | -24 | 60 |
|  | S1 |  | -54 | -18 | 45 |
|  | MCC |  | -6 | -15 | 45 |
|  | STG | 438 | -45 | -27 | 12 |
|  | RO |  | -51 | -18 | 15 |
|  | Insular |  | -33 | -9 | 12 |
|  | Putamen |  | -27 | -3 | -3 |
|  | Thalamus |  | -12 | -30 | 3 |
| C02 (R.MN) | M1 | 3557 | 51 | -9 | 45 |
|  | S1 |  | 27 | -36 | 63 |
|  | SMA |  | 9 | -21 | 57 |
|  | MCC |  | 9 | 3 | 45 |
|  | RO |  | 39 | -21 | 20 |
|  | Thalamus |  | 27 | -36 | 63 |
| C04 (aDMN) | mPFC | 4420 | -3 | 48 | -6 |
|  | ACC |  | -6 | 45 | -3 |
|  | SMG |  | 12 | 60 | 9 |
|  | Caudate |  | 18 | 24 | -3 |
| C05 (R.FPN) | MFG | 4286 | 45 | 18 | 45 |
|  | IFG |  | 51 | 27 | 18 |
|  | IPL | 1386 | 51 | -51 | 42 |
|  | AG |  | 33 | -57 | 45 |
|  | Supramaginal gyrus | | 57 | -39 | 45 |
|  | MTG |  | 66 | -42 | 6 |
| C06 (L.FPN) | IFG | 5534 | -42 | 12 | 24 |
|  | AG |  | -36 | -63 | 42 |
|  | IPL |  | -33 | -54 | 42 |
| C07 (SN) | ACC | 2339 | 0 | 36 | 24 |
|  | MCC |  | 3 | 21 | 36 |
|  | Caudate | 333 | 12 | 6 | 9 |
|  | Insular |  | 36 | 15 | 0 |
|  | Putamen |  | 24 | 9 | 3 |
|  | Thalamus |  | 9 | -21 | 0 |
|  | Caudate | 296 | -12 | 9 | 6 |
|  | Insular |  | -42 | 12 | -6 |
|  | Putamen |  | -27 | 6 | 3 |
| C08 (VN) | Calcarine gyrus | 2208 | 18 | -63 | 15 |
|  | Cuenus |  | 21 | -75 | 21 |
|  | MOG |  | 36 | -78 | 15 |
| C09 (vl.FPN) | IFG | 2502 | -45 | 24 | -12 |
|  | SMG |  | -3 | 48 | 45 |
|  | AG | 645 | -57 | -57 | 33 |
|  | Supramaginal gyrus | | -51 | -66 | 36 |
|  | MTG |  | -54 | -60 | 21 |
|  | IFG | 188 | 51 | 21 | -9 |
| C10 (DAN) | SPL | 2827 | -18 | -63 | 57 |
|  | Precuenus |  | -6 | -66 | 54 |
|  | SFG |  | -18 | -1 | 60 |
|  | SFG |  | 36 | 4 | 60 |
|  | IPL | 172 | -54 | -42 | 48 |
| C11 (RON) | STG | 2687 | 66 | -24 | 12 |
|  | Supramaginal gyrus | | 66 | -21 | 18 |
|  | RO |  | 63 | 6 | 15 |
|  | STG | 2497 | -57 | -9 | 6 |
|  | Supramaginal gyrus | | -63 | -27 | 24 |
|  | RO |  | -60 | 0 | 12 |
| C12 (IN) | Insular | 5343 | -39 | 6 | -3 |
|  | Insular |  | 42 | 15 | -6 |
| C13 (pDMN) | Precuneus | 2089 | 0 | -54 | 36 |
|  | AG | 555 | -42 | -57 | 45 |
|  | AG | 454 | 54 | -51 | 27 |
| C14 (DMN) | Precuneus | 1263 | -6 | -57 | 9 |
|  | PCC |  | -6 | -51 | 24 |
|  | mPFC | 859 | 3 | 60 | -3 |
|  | SMG |  | 0 | 63 | 6 |
|  | AG | 348 | -48 | -69 | 24 |
|  | AG | 195 | 48 | -63 | 24 |

Table S3. 13 networks estimated from ICA

**Supplementary Table 4**

| Network | Network | F | p |
| --- | --- | --- | --- |
| C01 (L.MN) | C02 (R.MN) | 10.218 | < 0.001 |
| C02 (R.MN) | C14 (DMN) | 3.17 | 0.05 |
| C04 (aDMN) | C09 (vL.FPN) | 4.189 | 0.024 |
| C04 (aDMN) | C12 (IN) | 6.313 | 0.005 |
| C08 (VN) | C12 (IN) | 3.547 | 0.04 |
| C08 (VN) | C14 (DMN) | 5.261 | 0.01 |
| C09 (vL.FPN) | C10 (DAN) | 3.588 | 0.039 |
| C10 (DAN) | C14 (DMN) | 3.179 | 0.05 |
| C11 (RON) | C14 (DMN) | 3.383 | 0.046 |
| C12 (IN) | C14 (DMN) | 7.964 | 0.002 |

Table S4. The group effect in the FNC between networks
